# Donor killer immunoglobulin-like receptor genes and reactivation of cytomegalovirus after HLA-matched hematopoietic stem-cell transplantation: HLA-C allotype is an essential cofactor

**DOI:** 10.3389/fimmu.2013.00036

**Published:** 2013-02-21

**Authors:** Carolyn E. Behrendt, Ryotaro Nakamura, Stephen J. Forman, John A. Zaia

**Affiliations:** ^1^Division of Biostatistics and Epidemiology, City of HopeDuarte, CA, USA; ^2^Department of Hematology and Hematopoietic Cell Transplantation, City of HopeDuarte, CA, USA; ^3^Department of Virology, Beckman Research Institute of the City of HopeDuarte, CA, USA

**Keywords:** cytomegalovirus, hematopoietic stem-cell transplantation, HLA, killer Ig-like receptors, natural killer cells

## Abstract

Natural killer (NK) cells whose killer immunoglobulin-like receptors (KIRs) recognize human leukocyte antigen (HLA) ligand are “licensed” for activity. In contrast, non-licensed NK cells display KIRs for which ligand is absent from the self genotype and are usually hyporesponsive. Surprisingly, non-licensed cells are active in tumor control after hematopoietic stem-cell transplantation (HSCT) and dominate NK response to murine cytomegalovirus (CMV) infection. From those reports, we hypothesized that control of human CMV early after HSCT is influenced by donor KIR genes whose HLA ligand is absent-from-genotype of HLA-matched donor and recipient. To investigate, we studied CMV reactivation through Day 100 after grafts involving CMV-seropositive donor and/or recipient. A multivariate proportional rates model controlled for variability in surveillance and established covariates including acute graft-versus-host disease; statistical significance was adjusted for testing of multiple KIRs with identified HLA class I ligand (*2DL1*, *2DL2/3*, *2DS1*, *2DS2*, full-length *2DS4*, *3DL1/3DS1*, *3DL2*). Among HSCT recipients (*n* = 286), CMV reactivation-free survival time varied with individual donor KIR genes evolutionarily specific for HLA-C: when ligand was absent from the donor/recipient genotype, inhibitory KIRs *2DL2* (*P* < 0.0001) and *2DL1* (*P* = 0.015) each predicted inferior outcome, and activating KIRs *2DS2* (*P* < 0.0001), *2DS1* (*P* = 0.016), and *2DS4* (*P* = 0.016) each predicted superior outcome. Otherwise, with ligand present-in-genotype, donor KIR genes had no effect. In conclusion, early after HLA-matched HSCT, individual inhibitory and activating KIR genes have qualitatively different effects on risk of CMV reactivation; unexpectedly, absence of HLA-C ligand from the donor/recipient genotype constitutes an essential cofactor in these associations. Being KIR- and HLA-C-specific, these findings are independent of licensing via alternate NK cell receptors (NKG2A, NKG2C) that recognize HLA-E.

## INTRODUCTION

Early after hematopoietic stem-cell transplantation (HSCT), the repertoire of natural killer (NK) cells in the recipient is less differentiated than in the donor, skewed toward expression of inhibitory receptor CD94–NKG2A and against expression of killer immunoglobulin-like receptors (KIR; Bjørklund et al., 2010). Within 8–12 weeks after HSCT, however, the proportion of KIR+ NK cells gradually normalizes through cell differentiation, and the responsiveness of KIR+ cells comes to depend on the presence of corresponding human leukocyte antigen (HLA) ligand (Haas et al., 2011). NK cell expansions in response to cytomegalovirus (CMV, either latent in the recipient or reactivated) preferentially express activating receptor NKG2C- and HLA-C-specific KIRs that have ligand present-in-genotype (Kuijpers et al., 2008; Della Chiesa et al., 2012; Foley et al., 2012a,b).

Under the tenets of NK cell licensing, those NK cells that express inhibitory KIRs specific for self HLA ligands are functionally competent (“licensed”; Orr and Lanier, 2010). Recent evidence indicates that licensing also applies to NK cells that express activating KIRs (Fauriat et al., 2010). Non-licensed NK cells, on the other hand, express inhibitory KIRs specific for HLA ligands that are absent from the self genotype. Typically, these non-licensed cells are hyporesponsive. Yet, early after HSCT, non-licensed NK cells are clinically beneficial: KIR ligand absent-from-genotype has been associated with lower incidence of leukemic relapse after HLA-matched HSCT (Hsu et al., 2005, 2006; Clausen et al., 2007; Miller et al., 2007) and with better progression-free survival after autologous HSCT for advanced neuroblastoma (Venstrom et al., 2009). According to a recent report (Orr et al., 2010), non-licensed cells dominate the NK response to murine CMV.

These published reports of NK cell functionality in the absence of HLA ligand raise the question of whether control of human CMV following HSCT can be mediated by donor KIRs whose ligand is absent-from-genotype shared by HLA-matched donor and recipient. To test this possibility, we prospectively followed HSCT recipients of grafts involving CMV-seropositive donor or recipient. Previous studies of the role of KIRs in controlling human CMV infection have mostly ignored the corresponding HLA ligands (Chen et al., 2006; Cook et al., 2006; Zaia et al., 2009; Gallez-Hawkins et al., 2011; Stern et al., 2011) or have studied the absence of ligand for an aggregate group of inhibitory or activating KIRs (Hadaya et al., 2008; Stern et al., 2008). In contrast, our study evaluated interactions between individual donor KIR genes and the presence or absence of ligand genes in the donor/recipient genotype, as risk factors for CMV reactivation through Day 100 after transplantation. Our analysis adjusted for the testing of multiple hypotheses and took into account established covariates, including method of CMV surveillance (Boeckh et al., 1997), serologic status of donor and recipient (Ljungman et al., 2010), onset of acute graft-versus-host disease (GVHD; Meyers et al., 1986; Bacigalupo et al., 1995; Takenaka et al., 1997; Osarogiagbon et al., 2000; Yanada et al., 2003; Walker et al., 2007; Zaia et al., 2009), use of sirolimus-based regimens for GVHD prophylaxis (Marty et al., 2007), and source of stem-cell graft (Guerrero et al., 2012).

## MATERIALS AND METHODS

### ETHICS STATEMENT

The protocol for this observational cohort study was approved in advance by the hospital’s institutional review board. Before undergoing HSCT, all subjects gave written informed consent to prospective follow-up and periodic blood sampling.

### SUBJECTS

Eligible subjects were consecutive patients age 18 or older who underwent a first HLA-matched, allogeneic HSCT in 2000–2006 for underlying hematologic disease, receiving a graft of unmanipulated stem-cells from the peripheral blood or bone marrow of a non-syngeneic sibling or unrelated donor. Further, only those transplants in which donor and/or recipient was CMV-seropositive prior to transplant were eligible for study. Exclusion criteria for the current analysis were incomplete data [on donor CMV serostatus (*n* = 5) or KIR genotype (*n* = 5)] or lack of standard CMV surveillance (due to reactivation detected immediately before transplantation or other reason [*n* = 5]). CMV prophylaxis was not utilized, but preemptive treatment with ganciclovir was started upon detection of CMV reactivation in blood as previously described (Zaia et al., 2009).

### TYPING OF HLA AND KIR

Human leukocyte antigen typing of donors and recipients was performed using one or more of the following methods: microbead array assay with sequence-specific oligonucleotide probes (One Lambda, Canoga Park, CA, USA), polymerase chain reaction (PCR) amplification with sequence-specific primers (Olerup SSP AB, Saltsjöbaden, Sweden; Invitrogen Corporation, Carlsbad, CA, USA), and sequence-based typing (Celera Corporation, Alameda, CA, USA). HLA-C allotypes belong to two groups distinguished by the amino acid residue at position 80; those allotypes with an asparagine are termed group 1 or *C1*, and those with a lysine are termed group 2 or *C2* (Vilches and Parham, 2002).

For KIR typing, a previously described method (Sun et al., 2004) was used to identify the functional, non-framework KIR genes (*2DS1-5*; *3DS1*; *2DL1-3* and *2DL5*; *3DL1*) and distinguish full-length from deleted alleles of *KIR2DS4* (Parham, 2005).

### SURVEILLANCE FOR CMV REACTIVATION

At physician discretion, surveillance for CMV usually began as close as possible to post-transplant Day 21 and generally continued until post-transplant Day 80–100. For surveillance, blood was collected once or twice-weekly and assayed by shell vial culture of whole blood (Gleaves et al., 1985). Whenever possible, plasma from the same specimen was assayed by DNA-quantitative PCR, limit of CMV detection: 200 genome copies/mL (Gallez-Hawkins et al., 2005). As clinically indicated, bronchoalveolar lavage and tissue biopsies were also tested for CMV by histopathology or tissue culture. The day of CMV reactivation was defined as the earliest positive result by any of these methods.

### STATISTICAL METHODS

The study endpoint was survival time free of CMV reactivation by Day 100 after HSCT. Follow-up was censored when Day 100 post-transplantation was reached or when CMV surveillance ended earlier. In addition, cumulative incidences of CMV reactivation and death without CMV were calculated as separate, competing events (Fine and Gray, 1999).

Because use of PCR and frequency of testing could vary during surveillance, it was necessary to consider each subject’s surveillance as one or more periods, with a new period being started whenever the conditions of surveillance changed, from twice-weekly to once per week or from testing by PCR and viral culture to testing by culture alone. To ensure that surveillance would be at least 1 day in duration even when CMV was detected in the subject’s first specimen, each surveillance period began on the eve of the first assay performed during that period. Multivariate analysis employed a proportional rates model of time to CMV reactivation or death; a robust sandwich covariance estimate took into account the intracluster dependence arising when there were multiple observations per subject (Lin et al., 2000).

The primary risk factors included the seven donor KIR genes with an identified HLA ligand: *KIR2DL1*, *KIR2DL2*, *KIR2DS1*, *KIR2DS2*, *KIR2DS4*, *KIR3DS1*, and universally present *KIR3DL2*. Each KIR gene was analyzed as: present with its evolutionarily specific ligand “present-in-genotype,” present with ligand “absent-from-genotype,” and KIR gene absent. For those KIRs that belong to pairs of alleles (*2DL2/2DL3* and *3DL1/3DS1*), one allele from each pair was selected for the multivariate analysis; no association was observed with the non-selected alleles when these were included also. Because of biological evidence of delayed expression of *KIR2DL1* (Fischer et al., 2007), the analysis considered whether associations with this KIR and with its homolog, *KIR2DS1*, were time-dependent. To do so, we considered delays of 0–8 weeks between day of transplantation and expression of these two KIRs; for each KIR, the delay with the best fit to the observed data was retained in the model. In order to limit the study’s overall Type I error rate to 5%, *P* values for the primary risk factors were adjusted for multiple hypothesis testing using Holm’s method, a more powerful version of the Bonferroni test (Holm, 1979).

The model was adjusted for potentially confounding time-dependent variables (onset of acute GVHD that ultimately reached grade II–IV (Przepiorka et al., 1995); twice-weekly versus once-weekly surveillance) and fixed covariates (donor’s CMV serostatus and sibling versus unrelated status; female-to-male graft; source of stem-cells; specific underlying disease; use of sirolimus for GVHD prophylaxis; year of transplantation). Additional factors considered in the model were the recipient’s CMV serostatus and age at transplantation, as well as donor KIR genes that have no identified HLA class I ligand (*2DS3*, *2DS5*, *2DL5*). Because hematologic diagnosis correlated so closely with intensity of conditioning regimen and disease status at transplantation, the latter two variables were dropped from multivariate analysis.

The assumption of proportionality of rates over time was verified by examining Schoenfeld residuals generated by the model. The fit of the model to individual subjects was assessed by examining dfbeta (weighted transformation of the score residual, approximating the change in a covariate’s effect when a given observation is omitted).

## RESULTS

### SUBJECTS

The cohort included 286 subjects [age 43.0(11.6) years] who underwent HLA-matched, allogeneic HSCT in which donor and/or recipient was CMV-seropositive (**Table 1**). Acute GVHD grade II–IV developed before or during follow-up in 154 (53.8%) subjects and was already present at start of surveillance in 70 (24.5%) subjects.

**Table 1:** Characteristics of HSCT recipients and grafts (*N* = 286)

	*N* (%)
**RECIPIENT**
Year of age at transplantation
18–39	103 (36.0)
40–59	160 (55.9)
60–68	23 (8.0)
Hematologic diagnosis
Leukemia	209 (73.1)
Acute lymphoid leukemia	53 (18.5)
Acute myeloid leukemia	91 (31.8)
Chronic lymphoid leukemia	3 (1.1)
Chronic myeloid leukemia	41 (14.3)
Myelodysplastic syndrome	21 (7.3)
Other	77 (26.9)
Hodgkin lymphoma	6 (2.1)
Multiple myeloma	4 (1.4)
Myeloproliferative disorder	7 (2.5)
Non-Hodgkin lymphoma	49 (17.1)
Severe aplastic anemia	11 (3.9)
Calendar year of transplantation
2000–2002	104 (36.4)
2003–2006	182 (63.6)
Prophylaxis for graft-versus-host disease	
Sirolimus and tacrolimus	50 (17.5)
Tacrolimus and methotrexate	66 (23.1)
Mycophenolate mofetil-based regimen	95 (33.2)
Cyclosporin A and methotrexate	75 (26.2)
**GRAFT**
Donor type	
Sibling	184 (64.3)
Unrelated	102 (35.7)
Source of graft	
Peripheral blood	239 (83.6)
Bone marrow	47 (16.4)
Sex of donor, recipient	
D female, R male	58 (20.3)
D female, R female	57 (19.9)
D male, R male	96 (33.6)
D male, R female	75 (26.2)
Pretransplant CMV serostatus of donor, recipient	
D-, R+	76 (26.6)
D+, R-	29 (10.1)
D+, R+	181 (63.3)

*CMV, cytomegalovirus; D, donor; HSCT, hematopoietic stem-cell transplant; R, recipient.*

Donor’s KIR genes, classified by presence or absence of HLA ligand when applicable, are shown in **Table 2**. Being in linkage disequilibrium, donor *KIR2DL2* and *KIR2DS2* were almost always present together, but occasionally one or the other gene appeared alone: 123 subjects had donors with *KIR2DL2*, of whom 120 had donor *KIR2DS2* also, and 123 subjects had donors with *KIR2DS2*, of whom 120 had donor *KIR2DL2* also.

**Table 2:** Donor KIR genes, by presence of HLA ligand in shared genotype of donor and recipient pair (*N* = 286).

	*N* (%)
**Inhibitory KIR**
*2DL1*	
With *C2*	162 (56.7)
Without *C2*	114 (39.9)
No donor *KIR2DL1*	10 (3.5)
	*2DL2*	
With *C1*	103 (36.0)
Without *C1*	20 (7.0)
No donor *KIR2DL2*	163 (57.0)
	*2DL3*	
With *C1*	225 (78.7)
Without *C1*	38 (13.3)
No donor *KIR2DL3*	23 (8.0)
	*2DL5*	
Present	138 (48.3)
Absent	148 (51.7)
	*3DL1*	
With *B-Bw4*	152 (53.2)
Without *B-Bw4*	118 (41.3)
No donor *KIR3DL1*	16 (5.6)
*3DL2* (universally present)	
With *A-3* and/or *A-11*	95 (33.2)
Without *A-3* and/or *A-11*	191 (66.8)
**Activating KIR**
*2DS1*	
With *C2*	61 (21.3)
Without *C2*	43 (15.0)
No donor *KIR2DS1*	182 (63.6)
*2DS2*	
With *C1*	105 (36.7)
Without *C1*	18 (6.3)
No donor *KIR2DS2*	163 (57.0)
*2DS3*	
Present	75 (26.2)
Absent	211 (73.8)
*2DS4* (full-length allele)	
With C*0501,C*1601	10 (3.5)
Without C*0501 or C*1601	121 (42.3)
No donor *KIR2DS4* (full-length allele)	155 (54.2)
*2DS5*	
Present	81 (28.3)
Absent	205
*3DS1*	
With *B-Bw4*	61 (21.3)
Without *B-Bw4*	46 (16.1)
No donor *KIR3DS1*	179 (62.6)

### SURVEILLANCE FOR CMV REACTIVATION

In nearly all (90.6%) subjects, surveillance began within the 7 days before or after Day 21 post-transplantation. Usually, surveillance was performed twice per week and utilized both viral culture and DNA-quantitative PCR. However, in 28.7% of subjects, at least some surveillance was by culture alone, and in 15.4% of subjects, at least some surveillance was once per week.

At the close of surveillance by Day 100 post-transplantation, cumulative incidences (standard deviation) of CMV reactivation and of death not preceded by CMV disease or reactivation were 66.4(3.0) and 3.0(1.0)%, respectively. There were seven cases of CMV disease [gastritis (*n* = 3), colitis (*n* = 2), or pneumonitis (*n* = 2)], none of which contributed to a death. Instead, deaths without prior CMV activity (*n* = 8) were attributed to acute respiratory distress syndrome and/or multi-organ system failure (*n* = 5), relapsed or progressive leukemia (*n* = 2), and veno-occlusive disease of the liver (*n* = 1). Of subjects (*n* = 96) who did not experience CMV reactivation or death, nearly all (86.5%) continued surveillance through at least Day 80 post-HSCT.

Because cases of CMV reactivation so thoroughly outnumbered deaths without prior reactivation (by a ratio of 182:8), the analysis of CMV reactivation-free survival closely approximated an analysis of time to CMV reactivation. Moreover, had the eight subjects who died without CMV reactivation been omitted from analysis, the results would have been virtually unchanged (data not shown).

### GENETIC ASSOCIATIONS WITH CMV REACTIVATION

In multivariate analysis (**Table 3**), five of the seven KIRs studied were associated with CMV reactivation, as follows. Inhibitory *KIR2DL2* and *KIR2DL1* were independently associated with increased likelihood of CMV reactivation, but only when the corresponding ligand was absent from the genotype shared by donor and recipient. Of note, the association with *KIR2DL2* was more marked and began earlier than the association with *KIR2DL1*, which did not begin until Day 49 post-HSCT. Likewise, activating *KIR2DS2*, *KIR2DS1*, and *KIR2DS4* (full-length allele) were each associated with reduced likelihood of reactivation, but only when the corresponding ligand was absent from the genotype. Of note, the association with *KIR2DS2* was more marked and began earlier than the association with *KIR2DS1*, which did not begin until Day 21. The protection against CMV reactivation associated with *KIR2DS4* (full-length allele), although significant, was less marked than that associated with *KIR2DS2* and *KIR2DS1*.

**Table 3:** CMV reactivation-free survival after HLA-matched, allogeneic HSCT (*N* = 286).

			Protection←Rate ratio→Hazard								
Risk factor	Rate ratio (95% CI)	*P*	0.05	0.1	0.2	0.5	1	2	5	10	20
**Donor inhibitory KIR:**
*KIR2DL2*
Without *C1*	7.97 (3.71, 17.1)	<0.0001						- - - - - -•- - - - - -		
With *C1*	1.17 (0.66, 2.09)	NS				- -°- -	
Absent	1.00		
^†^ *KIR2DL1*, starting Day 49 post-HSCT
Without *C2*	2.40 (1.39, 4.13)	0.015					- - -•- - -	
With *C2*	1.03 (0.60, 1.75)	NS					- -°- -
Absent	1.00
*KIR3DL2*
Without *A-3* and/or *A-11*	1.06 (0.78, 1.43)	NS					- -°- -
With *A-3* and/or *A-11*	1.00
**Donor activating KIR:**
*KIR2DS2*
Without *C1*	0.05 (0.02, 0.13)	<0.0001	-•- - - - - - - - -						
With *C1*	0.59 (0.34, 1.04)	NS					- -°- -
Absent	1.00
^†^ *KIR2DS1*, starting Day 21 post-HSCT
Without *C2*	0.41 (0.24, 0.71)	0.016			- - -•- - -	
With *C2*	0.68 (0.41, 1.16)	NS				- -°- -	
Absent	1.00
Full-length *KIR2DS4*
Without C*0501 or C*1601	0.61 (0.45, 0.83)	0.016				- - -•- - -	
With C*0501 or C*1601	0.63 (0.27, 1.49)	NS					- -°- - - -	
Absent	1.00
*KIR3DS1*
Without *B-Bw4*	1.33 (0.76, 2.32)	NS					- -°- -	
With *B-Bw4*	1.66 (1.10, 2.49)	NS						- -°- -	
Absent
**Covariates:**
^†^ Method of CMV surveillance, by Era	1.22 (0.61, 2.43)
Viral Culture plus PCR, 2000–2002	1.00
Viral Culture Alone, 2000–2002	3.15 (1.59, 6.25)
Viral Culture plus PCR, 2003–2006	0.29 (0.10, 0.89)
Viral Culture Alone, 2003–2006	1.22 (0.61, 2.43)
^†^ Frequency of CMV surveillance	
Twice per week	1.27 (0.77, 2.11)
Once per week	1.00
^†^ Acute GVHD	
Had occurred	1.73 (1.27, 2.36)
Had not occurred	1.00
CMV serostatus and type of donor	
Seronegative sibling donor	2.00 (1.19, 3.37)
Seropositive sibling donor	1.00
Seronegative unrelated donor	0.99 (0.66, 1.48)
Seropositive unrelated donor	1.07 (0.74, 1.57)
GVHD prophylaxis	
Sirolimus and tacrolimus	0.37 (0.22, 0.60)
Other regimen	1.00
Female-to-male graft	
Yes	1.34 (0.93, 1.92)
No	1.00
Leukemia/myelodysplastic syndrome	
Yes	0.75 (0.54, 1.04)
No	1.00
Source of stem-cell graft	
Bone marrow	0.72 (0.47, 1.11)
Peripheral blood	1.00

*Solid circles illustrate statistically significant associations, while open circles illustrate non-significant ones. Only the primary risk factors were evaluated for statistical significance and illustrated graphically. Covariates were included if they improved the fit of the model to the data. *P* values were corrected for multiple hypothesis testing using the Holm–Bonferroni adjustment (Holm, 1979).*

† *Covariate’s effect is time-dependent, due to a change in a subject’s surveillance or to delayed onset of the risk factor*.

Donor KIR genes without identified HLA class I ligand (*KIR2DS3*, *KIR2DS5*, *KIR2DL5*) did not contribute to the fit of the model and were therefore omitted. When the model was limited to the primary risk factors, adjusted only for whether surveillance for CMV included PCR, the results were similar (data not shown). Whether a KIR’s ligand was present in one or two gene copies did not alter any of the associations (data not shown).

## DISCUSSION

According to this study, the risk of CMV reactivation following HLA-matched HSCT is associated with individual donor KIR genes, but only when their evolutionarily specific HLA-C ligand is absent from the shared donor/recipient genotype. Our results add to the evidence that non-licensed NK cells can play an important role in host control of tumors (Hsu et al., 2005, 2006; Clausen et al., 2007; Miller et al., 2007; Venstrom et al., 2009) and infection, specifically murine CMV (Orr et al., 2010), with one important difference. Unlike previous reports, our study suggests that “presumably non-licensed” NK cells (inhibitory KIRs with ligand absent-from-genotype) are associated with worse, not better, clinical outcome, while comparable activating KIRs (those with ligand absent-from-genotype) have qualitatively opposite effects, being associated with better, not worse, clinical outcome among our subjects. Also, unlike studies *in vitro* (Kim et al., 2008; Fauriat et al., 2010), our clinical study did not detect a dose effect from the gene copy number of HLA ligand.

The chief limitation of our study is the lack of *in vitro* data, on KIR expression in NK and T cells and on the licensing status of KIR-bearing cells early after allogeneic HSCT. Conclusions can still be drawn from these genotyping data, because a ligand absent-in-genotype can be expected to be absent in expression also. Although unstudied here, expression of NKG2A and NKG2C could not have confounded our results: because our findings are KIR- and HLA-C-specific, they are independent of licensing through NKG2A or NKG2C, which recognize HLA-E.

The current study is not the first to associate the control of human CMV with KIRs that are evolutionarily specific for HLA-C (Kuijpers et al., 2008; Foley et al., 2012a,b). Such KIRs present a number of special considerations. For instance, *KIR2DL2* and *KIR2DS2* are in linkage disequilibrium; as a result, our sample included few donors having one of these genes but not the other (“discordant donors,” *n* = 6). Nevertheless, associations with each of these two KIRs were so strong that they could be detected in our sample. Current findings do not depend on the six subjects with discordant donor, however. When those subjects are omitted, the model still shows that, relative to having neither *KIR2DL2* nor *KIR2DS2*, having both donor genes with ligand absent-from-genotype is associated with a 0.4-fold reduction in risk of reactivation, a figure that corresponds, as it should do, to the product of the associations we report in **Table 3** for *KIR2DL2* without *C1* (7.97) and *KIR2DS2* without *C1* (0.05). In other words, in the absence of ligand, the combined effects of inhibitory *KIR2DL2* and activating *KIR2DS2* result in a net reduction in CMV reactivation, the 20-fold reduction in risk associated with *KIR2DS2* (rate ratio 0.05) outweighing the eightfold increase in risk associated with *KIR2DL2.* Thus, despite their partial homology, inhibitory *KIR2DL2* and activating *KIR2DS2* have qualitatively different effects on risk of CMV reactivation, as *KIR2DL1* and *KIR2DS1* currently do also.

*KIR2DS2* has reportedly lost avidity for its evolutionary ligand, *C1* (Moesta et al., 2010). Nevertheless, in the current study (**Table 3**), *C1* in genotype eliminated the benefit associated with *KIR2DS2* just as it eliminated the disadvantage associated with *KIR2DL2*. Our genotypic data cannot reveal the mechanism underlying this apparent *KIR2DS2*–*C1* interaction, but the finding suggests that, at least in the setting of CMV infection after HSCT, binding affinity of *KIR2DS2* for *C1* may be present *in vivo*, perhaps facilitated by a peptide of viral origin. *C1*-specific KIRs (*KIR2DL2*, *KIR2DL3*) reportedly recognize a few *C2* alleles also, most strongly C*0501 (Graef et al., 2009). If the latter allele is included as a ligand for *KIR2DL2* (and for its partial homolog, *KIR2DS2*), the results of the current study are virtually unchanged (data not shown). In addition, *KIR2DS4* recognizes more than one ligand, chiefly a *C2* allele (C*0501), a *C1* allele (C*1601), and A*1102 (an allele not present among our sample; Graef et al., 2009). In our study, because the latter three ligands are uncommon, *KIR2DS4* was nearly always present without ligand-in-genotype, potentially explaining a previously reported association between *KIR2DS4* and reduced risk of CMV reactivation after HSCT (Chen et al., 2006; Cook et al., 2006; Zaia et al., 2009; Gallez-Hawkins et al., 2011; Stern et al., 2011).

Because *KIR2DL2* and *KIR2DL3* are alleles, it is possible for one or both alleles to be present in a given donor. In our analysis, the association with donor *KIR2DL2* was unaffected by whether *KIR2DL3* was also present (data not shown), a finding consistent with reports that *KIR2DL2* is a stronger receptor for ligand *C1* than is *KIR2DL3* (Moesta et al., 2008, 2010; Fadda et al., 2010). Furthermore, during NK cell development from progenitor cells, *KIR2DL1* appears later and at lower frequency than *KIR2DL2* and *KIR2DL3*, which dominate the early NK cell repertoire (Fischer et al., 2007). Accordingly, current associations of CMV reactivation with *KIR2DL1* and its partial homolog, *KIR2DS1*, were qualitatively similar to, but with later onset and lower magnitude than, the associations of their non-time-dependent counterparts, *KIR2DL2* and *KIR2DS2*.

Under the arming model (Raulet and Vance, 2006), NK cells are hyporesponsive by default and become responsive upon interaction between inhibitory KIR and ligand. In contrast, under the disarming model (Raulet and Vance, 2006), NK cells are responsive by default and become hyporesponsive from *lack* *of* interaction between inhibitory KIR and ligand. Our own observations are more consistent with the latter model, with the difference that, in our study, disarming applies to activating as well as inhibitory KIRs, which in turn exert qualitatively different effects on control of CMV after HLA-matched HSCT. Specifically, inhibitory KIR without HLA interaction results in *inferior* outcome (greater incidence of CMV reactivation), while activating KIR without HLA interaction results in *superior* outcome (lower incidence of reactivation) among our subjects.

Taken together, our observations suggest that, at least in the first 100 days after HLA-matched HSCT, the HLA ligand, rather than providing a license for function, serves as a gatekeeper of inherent NK cell activity: with ligand, KIR-mediated activity is held in check, while without ligand, KIR-mediated activity is unrestricted. Moreover, this “gatekeeping” applies to inhibitory and activating KIRs alike, the only difference being that inhibitory KIR’s inherent activity is harmful to host control of CMV, while activating KIR’s effect is beneficial, apparently promoting viral clearance. It is possible that this gatekeeping concept can be exploited in the development of NK-based cell therapy or in predicting a transplant recipient’s risk of CMV reactivation.

## Conflict of Interest Statement

The authors declare that the research was conducted in the absence of any commercial or financial relationships that could be construed as a potential conflict of interest.
